# BASIL: A toolbox for perfusion quantification using arterial spin labelling

**DOI:** 10.1162/imag_a_00041

**Published:** 2023-12-05

**Authors:** Michael A. Chappell, Thomas F. Kirk, Martin S. Craig, Flora A. Kennedy McConnell, Moss Y. Zhao, Bradley J. MacIntosh, Thomas W. Okell, Mark W. Woolrich

**Affiliations:** Sir Peter Mansfield Imaging Center, School of Medicine, University of Nottingham, Nottingham, United Kingdom; Wellcome Centre for Integrative Neuroimaging, Nuffield Department of Clinical Neurosciences, University of Oxford, United Kingdom; Quantified Imaging, London, United Kingdom; Department of Radiology, Stanford University, Stanford, CA, United States; Hurvitz Brain Sciences Research Program, Sunnybrook Research Institute, University of Toronto, Toronto, ON, Canada; Department of Medical Biophysics, Faculty of Medicine, University of Toronto, Toronto, ON, Canada; Oxford Centre for Human Brain Activity (OHBA), Wellcome Centre for Integrative Neuroimaging, Department of Psychiatry, University of Oxford, Warneford Hospital, Oxford, United Kingdom

**Keywords:** arterial spin labelling, perfusion, cerebral blood flow, arterial transit time, variational Bayesian inference

## Abstract

Arterial Spin Labelling (ASL) MRI is now an established non-invasive method to quantify cerebral blood flow and is increasingly being used in a variety of neuroimaging applications. With standard ASL acquisition protocols widely available, there is a growing interest in advanced options that offer added quantitative precision and information about haemodynamics beyond perfusion. In this article, we introduce the BASIL toolbox, a research tool for the analysis of ASL data included within the FMRIB Software Library (FSL), and explain its operation in a variety of typical use cases. BASIL is not offered as a clinical tool, and nor is this work intended to guide the clinical application of ASL. Built around a Bayesian model-based inference algorithm, the toolbox is designed to quantify perfusion and other haemodynamic measures, such as arterial transit times, from a variety of possible ASL input data, particularly exploiting the information available in more advanced multi-delay acquisitions. At its simplest, the BASIL toolbox offers a graphical user interface that provides the analysis options needed by most users; through command line tools, it offers more bespoke options for users needing customised analyses. As part of FSL, the toolbox exploits a range of complementary neuroimaging analysis tools so that ASL data can be easily integrated into neuroimaging studies and used alongside other modalities.

## 1 Introduction

Arterial Spin Labelling MRI is now an established and increasingly widely used method for non-invasively imaging cerebral perfusion^1^. BASIL is a toolbox for the quantification of perfusion and other haemodynamic parameters from Arterial Spin Labelling (ASL) MRI data. Its speciality is robust precision quantification using Bayesian inference methods, and it is equally well-suited to both standard single-delay acquisitions as recommended by the consensus paper (Alsop et al., 2015), and advanced multi-delay acquisitions that better sample the kinetics of the ASL tracer (Woods et al., 2023). Critically, BASIL approaches the analysis of all ASL data using the same model and algorithm, allowing for consistency and correspondence to be achieved between studies irrespective of the acquisition scheme employed. Since the analysis can be applied to every common form of ASL, it is possible to process data acquired from any MRI vendor’s platform, and to this end BASIL has been used with all the major product sequences and many widely used research sequences.

**Fig. 1.**
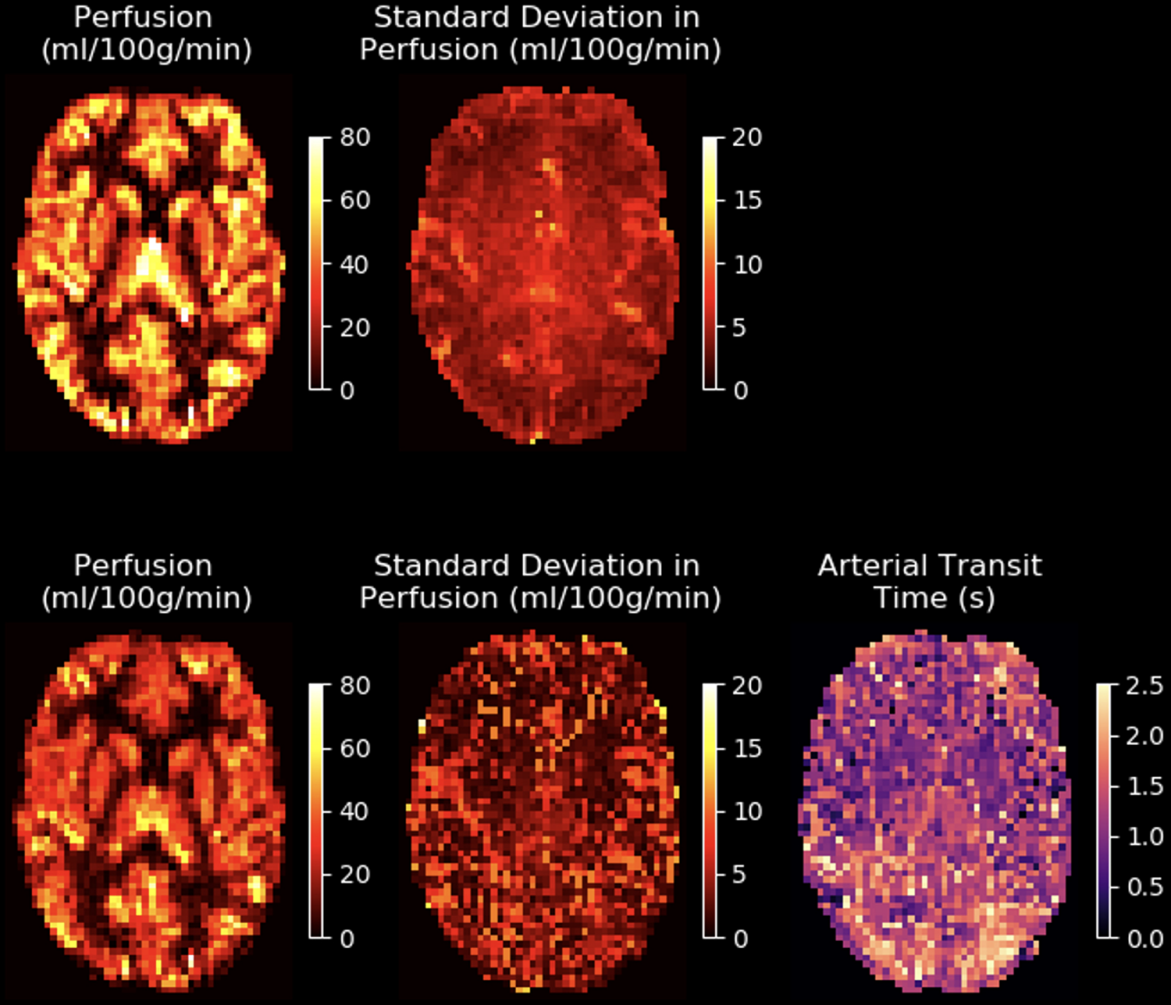
Example quantified perfusion images from single-delay (top row) and multi-delay (bottom row) ASL in a single subject. Also shown is the estimated uncertainty in the perfusion parameter, given as the estimated standard deviation on the perfusion value in the voxel, and the corresponding ATT estimates for the multi-delay data. The single-delay data have been fitted in “white paper” mode, following the assumptions of the ASL consensus paper. Due in particular to the assumption that ATT = 0 s, the resultant perfusion estimates are higher than in the multi-delay case. In the multi-delay case, fitting an extra parameter (ATT) increases the uncertainty in perfusion estimates for some voxels, leading to a more variable standard deviation map for this parameter.

An example of BASIL’s output operating on single- and multi-delay pseudo-continuous ASL data is shown in Figure 1, showing both perfusion and (for the multi-delay case) arterial transit time (ATT) estimates. Also shown is the estimated uncertainty on the perfusion values, given as a standard deviation.

The BASIL toolbox is distributed as part of the FMRIB Software library (FSL, www.fmrib.ox.ac.uk/fsl) (Jenkinson et al., 2011; Woolrich et al., 2009), a version having first been offered in FSL v5.0.1 in 2012; this paper refers to the latest version in FSL v6 (6.0.6^2^). The toolbox is provided under the same licence terms as FSL itself (free for academic use, https://fsl.fmrib.ox.ac.uk/fsl/fslwiki/Licence) and the code is open source (available at https://github.com/physimals). A review of the literature in 2023 indicates that it has been used in over 100 published studies^3^. Components of BASIL are also available as plug-ins for other neuroimaging and physiological imaging software tools, including ExploreASL (Mutsaerts et al., 2020) (an SPM-compatible ASL analysis tool, www.exploreasl.org) and Quantiphyse (a python-based graphical user interface for the analysis of physiological imaging data designed for non-expert users; www.quantiphyse.org). The Open Science Initiative for Perfusion Imaging has created an ASL Pipeline Inventory which compares the features and requirements of currently available ASL tools, including the BASIL toolbox^4^.

**Fig. 2.**
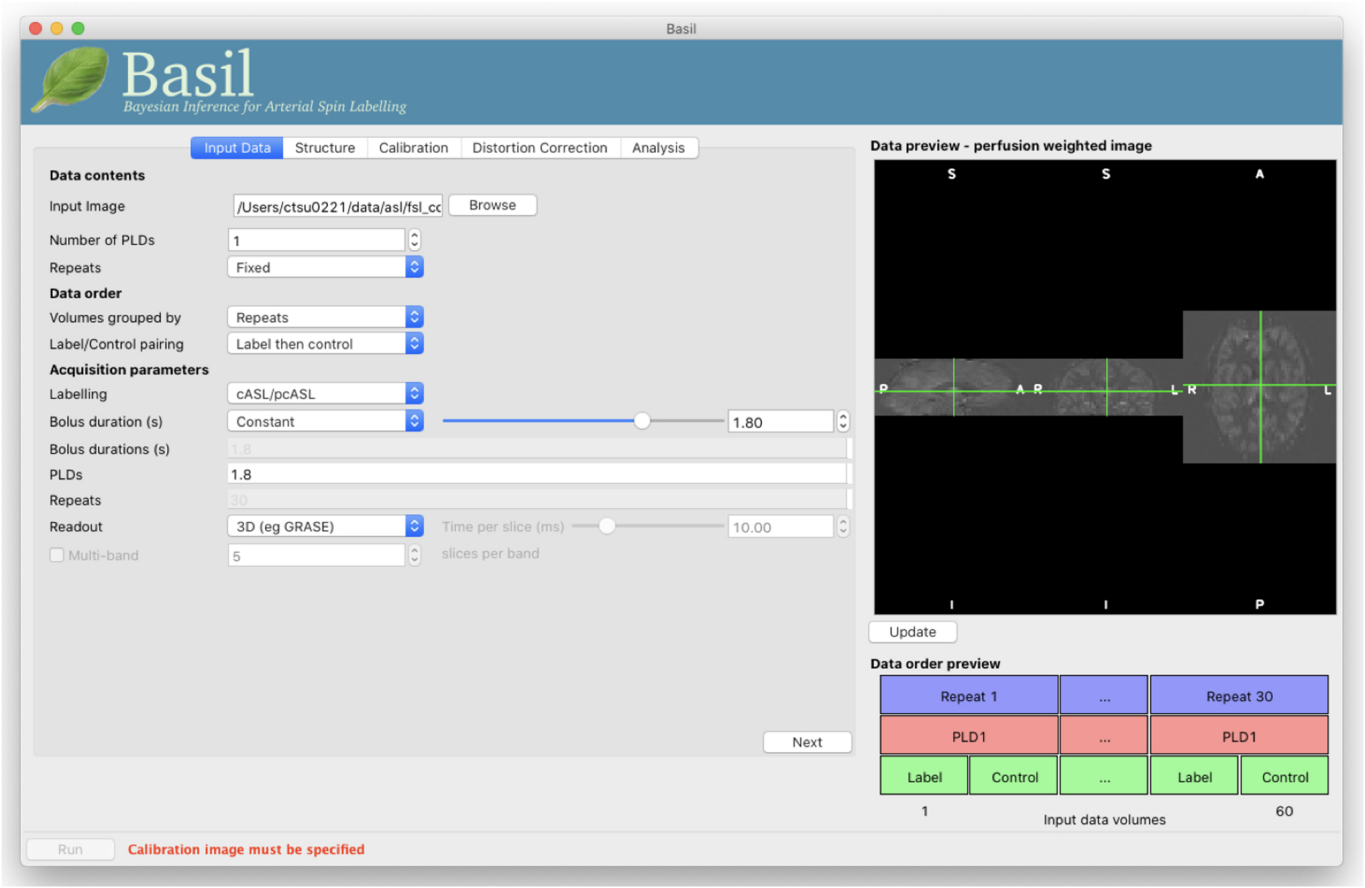
BASIL toolbox GUI (asl_gui) showing “Input data” tab loaded with single-delay PCASL after a preview of the label-control subtraction has been requested.

The BASIL toolbox includes a graphical user interface (GUI, asl_gui) that presents the main functionality that many users would require to analyse individual subject ASL data, as well as integration with other FSL tools to prepare data for group analysis. The toolbox is supported by online documentation and hands-on tutorial guides at https://asl-docs.readthedocs.io/en/latest/. The GUI, shown in Figure 2, directly interacts with the oxford_asl command line tool, the main command line interface to the toolbox. This link between GUI and command line allows analysis to be set up first in the GUI, but the associated command line call reused and adapted to create a batch script for processing large datasets. The design of the GUI follows the principle that 20% of capabilities will be sufficient for 80% of users; more advanced control is offered either via the oxford_asl command line tool or through the use of individual component tools for complete control over all analysis steps (as detailed in section 4). Notably, the basil command line tool itself is the interface to the kinetic model inference algorithm, which includes a range of kinetic models.

The aim of this paper is to describe the technology and functionality of the BASIL toolbox, documenting important details of the implementation in order to provide a high degree of transparency for its use. This work assumes background knowledge of ASL and those who are new to the modality are referred to resources such as Chappell et al. (2017) or https://asl-docs.readthedocs.io/en/latest/ for an introduction to both acquisition and analysis. Neither does this work attempt to guide users on how to choose between the various analysis strategies that are possible using the toolbox, particularly in the context of clinical applications. Such discussion can be found elsewhere (including but not limited to Alsop et al., 2015; Chappell et al., 2017; Jezzard et al., 2017; Pinto et al., 2020; Zhao et al., 2017).

## 2 Overview of ASL Analysis

Generating a perfusion-weighted image from ASL data is trivial, involving the subtraction of label and control images. Typically, multiple images will have been acquired to improve signal-to-noise ratio and an average is generally taken over all subtraction pairs. Two further steps are then required for quantification: 1) kinetic model fitting, to account for the relationship between signal intensity and delivery of labelled blood water via perfusion; 2) calibration, to relate signal intensity to absolute perfusion, scaling for the apparent concentration of the tracer via the equilibrium magnetisation of arterial water.

The ASL consensus paper (Alsop et al., 2015) combines these steps into a single process, given in terms of an equation that converts from raw image intensities to absolute perfusion using a proton density-weighted M0 image. For more advanced quantification, kinetic model fitting is achieved fitting a non-linear kinetic model (Buxton et al., 1998) to the data, which allows for correction of confounding haemodynamic effects and/or estimation of other haemodynamic information, such as arterial transit time (ATT), and arterial blood volume (aBV) in larger arteries (the “macrovasculature”) (Chappell et al., 2010; Petersen et al., 2006). Various strategies for calibration can be employed, reflecting both the type of M0 images available, and whether a reference region is used to estimate the magnetisation of arterial blood or if this is done on a voxelwise basis (Pinto et al., 2020).

As with other functional imaging modalities, correction for motion and distortion is possible for ASL perfusion images. Additionally, registration to a template space, for example, MNI152 “standard” space (Grabner et al., 2006), is often desirable as part of a study. All of these processes follow techniques used in other neuroimaging modalities, the closest being BOLD fMRI, but with particular challenges associated with the characteristics of ASL data.

## 3 The Basil ASL Analysis Pipeline

**Fig. 3.**
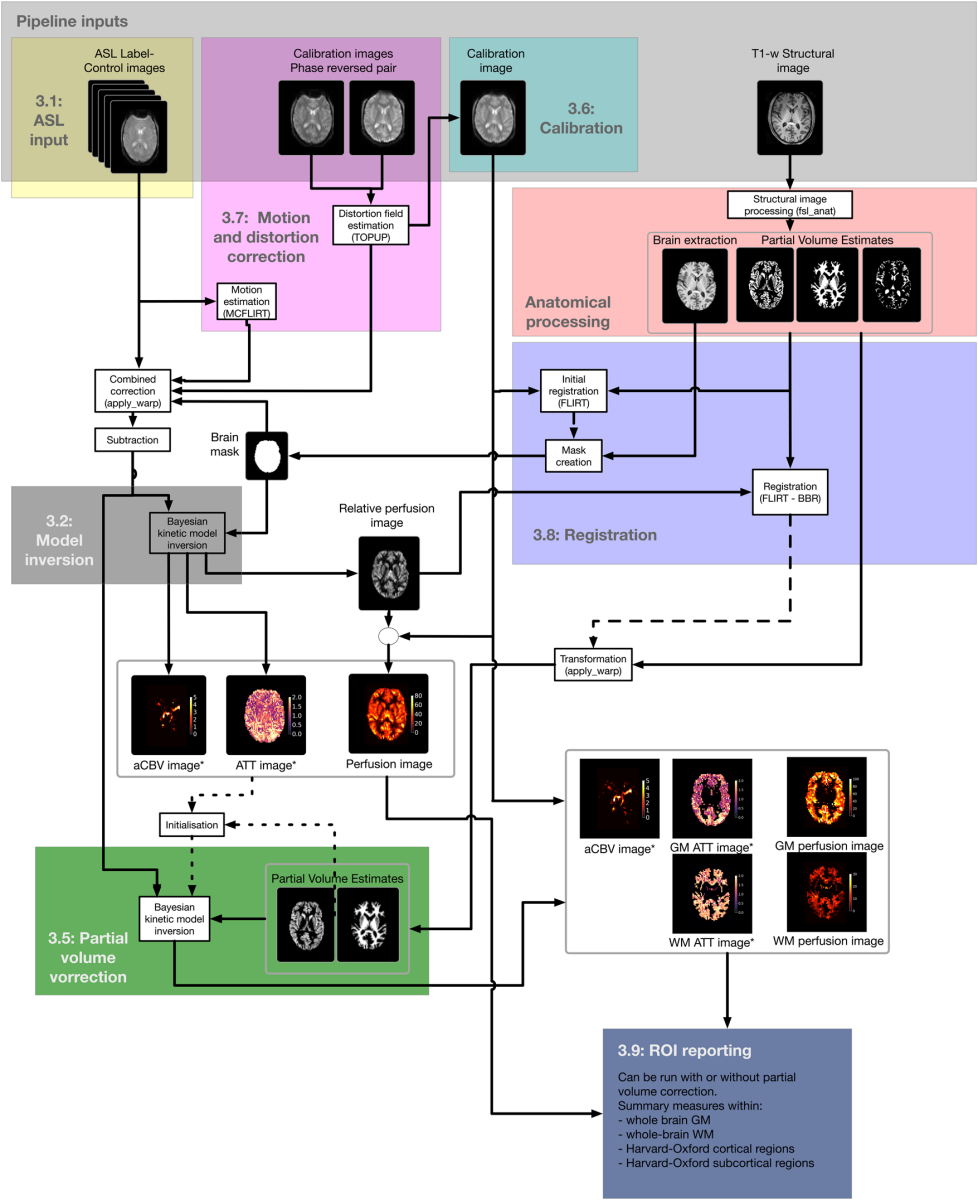
A graphical representation of the processing steps required to produce a perfusion image (scaled into absolute units) along with ATT. The numbers 3.1, 3.2 etc. refer to section headings in the manuscript text. Optional steps such as motion/distortion correction and partial volume correction have been included. * denotes an output for which multi-delay data are required.

The BASIL toolbox offers a complete analysis pipeline for ASL data that aims to cover the majority of use cases and will be discussed in the following section. The pipeline is accessed via either the toolbox GUI asl_gui or the command line tool oxford_asl, and in either case the underlying processing is the same (namely, using the individual components of the toolbox listed in section 4). Figure 3 shows a schematic diagram of the operations performed to process ASL data to obtain perfusion and ATT maps. If required, a user may perform the operations in a different order or with different settings by using the individual components of the toolbox listed in section 4. Consistent with the wider FSL toolbox, BASIL has been developed for research use and it has not been validated for clinical applications.

### 3.1 ASL data input

The tools in the BASIL toolbox all accept data in NIFTI format (consistent with the wider FSL tools). Users are recommended to convert data from DICOM to NIFTI using the widely-used dcm2niix tool^5^ which has compatibility with a range of ASL implementations. In the future, the emergence of tools that meet the recently adopted ASL-BIDS standard will facilitate easier conversion of DICOM to NIFTI data whilst preserving acquisition parameters from the DICOM header (Clement et al., 2022), though currently BASIL does not interface with ASL-BIDS. Since ASL can produce quantitative maps, it is important that conversion is done respecting scale-slopes stored in the DICOM header.

ASL data are processed in the native acquisition voxel grid, after having applied optional distortion and motion corrections (which do not alter the voxel grid, but rather transform data within the same grid). This choice is made on the basis that ASL is typically of low SNR and low spatial resolution, which implies high partial volume effects (discussed in section 3.5). Resampling the data onto a different voxel grid introduces interpolation artefacts that degrade data quality and negatively impact perfusion estimation (Kirk, 2021). Analysing the data in the native voxel grid as opposed to an anatomical or standard space is desirable because it minimises the amount of resampling or interpolation that is applied to the data; this is a point of difference with some other pipelines that do transform data^6^.

### 3.2 Kinetic model fitting

The distinctive feature of the BASIL toolbox is estimation of perfusion and other haemodynamic parameters through the approach taken to fitting of the kinetic model to estimate physiological parameters. For all types of ASL label-control data, the toolbox uses a fast Variational Bayesian inference algorithm to perform iterative non-linear kinetic model fitting, typically in a matter of minutes (Chappell et al., 2009). The use of Bayesian inference allows for the incorporation of prior information into the analysis, which assists robust parameter estimation in the presence of noise, particularly when several parameters are being estimated. Within the Bayesian framework, each parameter is treated as a component of a multi-variate normal distribution, for which the mean represents the most likely estimate and the variance gives a measure of uncertainty. Covariances between parameters retain their usual meaning, and all distribution parameters are estimated voxelwise from the complete data (all of the individual label-control pairs are exploited). The use of variance to represent parameter uncertainty is akin to a confidence interval (and allows the computation of confidence intervals if required); but it is not the same as the variance within a population (which cannot be inferred from a single subject’s data). Figure 1 shows example parameter maps from BASIL, where the variance has been converted to the standard deviation.

Each parameter is associated with a prior distribution, which can be distributional or spatial. A distributional prior is specified in terms of a normal distribution with mean and variance, which regularises parameter estimation and reflects the information known about the parameter before any data are seen (derived, e.g., from population studies). Bayesian inference can be viewed as an updating process, whereby the prior distribution is refined given the information available from the data. The default priors used by the toolbox are documented in the Supplementary Material. The prior distributions can be thought of as soft constraints, as opposed to the hard limits often implemented in non-probabilistic fitting algorithms. Values for the prior means have been derived from existing literature where possible (e.g., T1 values which assume a 3 T field strength) or are fairly typical given normal applications of ASL (e.g., ATT values based on typical labelling plane location). Prior standard deviations have been chosen to not unduly constrain the inference process by comfortably covering a range of plausible expected values.

The parameters of the distributional priors can be adjusted by the user via the command line interface for advanced analyses, for example, where a patient population is known to have different T1 values from the general population. In particular, the model fitting can optionally incorporate a map of subject-specific tissue T1 values in the same voxel grid as the ASL data, where these data have been collected. In this scenario, the default prior variance on T1 is retained to reflect measurement error in these values.

**Fig. 4.**
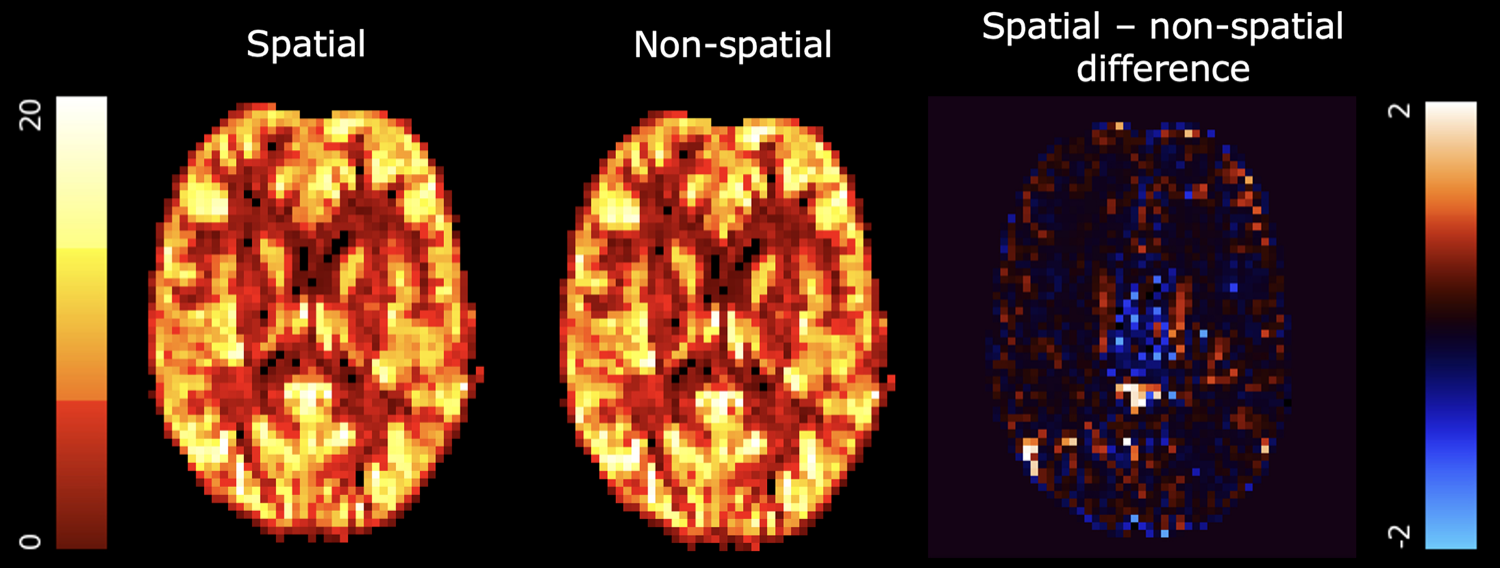
Perfusion maps derived from single-delay pseudo-continuous ASL with (left) and without (centre) spatial regularisation. The difference image is shown on the right. Quantities are in arbitrary units (i.e., non-calibrated data).

The alternative type of prior available within BASIL is a spatial prior, used to enable spatial regularisation which is recommended to improve the quality perfusion estimation (Groves et al., 2009; Penny et al., 2004). In contrast to the common usage of the term “spatial prior” in neuroimaging, this does not encode any particular belief about the value of a parameter at different locations within the brain. Instead, the spatial prior encodes the belief that parameter values should not vary greatly between neighbouring voxels on the same slice (the prior operates in the *xy* plane but not along the *z* axis to account for the large slice thickness typically used in ASL acquisitions). This provides a form of adaptive spatial regularisation on the estimated perfusion image, whereby the regularisation is driven by the confidence of parameter estimates in neighbouring voxels. Thus, where the data are of higher quality and there is higher confidence in the voxelwise estimates, there is less influence of neighbouring voxels and less apparent smoothing. This is preferable to conventional spatial smoothing of the ASL data as a preprocessing step before model fitting, since that involves the selection of an arbitrary smoothing parameter (e.g., full-width half maximum, FWHM) and can, for multi-delay data, lead to errors due to mixing of voxels with different (non-linear) kinetics (Groves et al., 2009). By contrast, no user-selected smoothing parameter is required to use spatial regularisation in BASIL. An important consideration for application of the spatial prior is that it operates on the voxel grid on which the ASL data are represented, but this may not be the voxel grid on which the data were acquired (e.g., some 3D GRASE sequences are interpolated after acquisition). In either case, the underlying assumption of the spatial prior remains valid (a local smoothness constraint), though the extent of regularisation may vary. The operation of the spatial prior is illustrated in Figure 4.

The use of Bayesian inference allows the same algorithm to be used for all data, that is, single- and multi-delay. Where a given parameter cannot be estimated from the data, the priors in BASIL provide a default value for these parameters, along with a reflection of the uncertainty that accrues due to them not being estimable. For example, since it is not possible to estimate ATT voxelwise from single-delay data, a BASIL analysis will take the prior mean as the value of this parameter and the prior variance over ATT will be reflected in the estimated confidence in the final perfusion estimate, meaning that it reflects the variability introduced by accounting for the lack of knowledge of ATT.

Inference proceeds in multiple stages to achieve good convergence to a global solution and thus a robust estimation of the parameters, following good practice in non-linear model fitting. In the first stage, only perfusion and ATT are inferred. Subsequent stages widen the range of parameters that are estimated, using the values from the previous stage for initialisation (in all cases, the priors remain the same). Only at the final stage are spatial priors applied to perfusion to implement spatial regularisation, using the full set of estimated parameters from an analysis without spatial priors as initialization. Table 1 documents the complete sequence of steps that are possible; the actual steps performed depend upon the analysis options chosen.

**Table 1.** Multi-step analysis process for kinetic model inference in BASIL.

Step	Parameters inferred			
	Tissue (grey matter**)	Macrovasculature	Labelled bolus (arterial input function)	White matter
1 Tissue	Perfusion, ATT			
2 Macrovascular correction	Perfusion, ATT	aCBV, BAT		
3 Label duration correction*	Perfusion, ATT	aCBV, BAT	LD	
4 Advanced kinetics (correction for dispersion and label exchange)	Perfusion, ATT, exchange parameter(s)	aCBV, BAT	LD, dispersion parameter(s)	
5 Correction for variable T1	Perfusion, ATT, exchange parameter(s), T1t	aCBV, BAT	LD, dispersion parameter(s), T1b	
6a Spatial regularisation	Spatial prior applied to perfusion parameter			
6b Partial volume effect correction with spatial regularisation	Perfusion**, ATT, exchange parameter(s), T1gm	aCBV, BAT	LD, dispersion parameter(s), T1b	Perfusion, ATT, exchange parameter(s), T1wm
	Spatial prior applied to GM and WM perfusion parameter			

Inference proceeds with only a subset of parameters being inferred at each step, with more parameters being progressively added. The table shows a complete analysis with all possible parameters (or parameter groups); analyses that do not require inference of all parameters can be processed in fewer steps, missing out those not required. Parameter values inferred in one step are used to initialise these same parameters in the subsequent step, the priors remain the same for all steps (excepting the introduction of the spatial prior in the final step if requested). ATT = Arterial Transit Time, BAT = Bolus Arrival Time, LD = Label Duration, aCBV = arterial Cerebral Blood Volume, T1x = T1 of x, where x is one of t = tissue, b = (arterial) blood, gm = grey matter, wm = white matter.

* This is primarily included for use with pulsed ASL when no further control has been made for the label duration.

** Tissue parameters from previous steps are taken to initialise the GM parameters for PVEc step. Initial values for GM and WM perfusion are set based on a ratio of 2.5:1 and scaled by the respective GM and WM PV estimates.

As part of the inference process, the influence of noise on the data is explicitly estimated in terms of the magnitude of the assumed white noise on the data in each voxel (defined as the precision of the Gaussian likelihood distribution on the measured data values)^7^. The noise parameter influences the degree to which prior information is used to inform the parameter estimates, as well as contributing to the resulting confidence in the parameter estimates. By default, the prior on the noise parameter is set to be uninformative, with the noise parameter being determined from the data. For datasets with fewer than five volumes (or if the user requests it), a more informative prior is employed that assumes an approximate SNR of 10, although ultimately the noise parameter is still estimated from the data where possible. This is further discussed in the first section of the Supplementary Material.

### 3.3 Kinetic models

The kinetic model implemented in the BASIL toolbox follows the general kinetic model as described in detail by Buxton et al. (1998). Pulsed (PASL), pseudo-continuous (PCASL), and Hadamard time-encoded labelling schemes are supported (the latter requires a prior decoding step that can be performed by asl_file). The modular nature of the codebase means that new variants of ASL can be incorporated without re-writing other parts of the toolbox; for example, this means velocity-selective ASL (Qin et al., 2022) may be supported in a future release.

By default, a “box-car” function is assumed for the arterial input function (AIF) with T1 decay at blood T1-rate, and a well-mixed single compartment with venous outflow assumed for the residue function with a distinct tissue T1. A range of alternative AIF (Chappell, Woolrich, Kazan, et al., 2013; Hrabe & Lewis, 2004) and residue functions (Lawrence et al., 2000; Parkes, 2005) are also available (see in the Supplementary Material), allowing modelling of effects, including dispersion and water exchange between capillary blood and extravascular space.

The default approach (i.e., when using the GUI or oxford_asl) is to perform kinetic model fitting independently of the calibration so that the calibration can be revisited later without needing to repeat the fit. For some combinations, the convolution of AIF and residue function is implemented analytically (using the formulation in Hrabe & Lewis (2004) for the default case), otherwise numerical convolution (trapezium rule with a resolution of 0.1 s) is used, which increases the processing time. The toolbox GUI includes the option to check whether the analysis to be performed matches (is “compliant with”) that specified in the consensus paper (Alsop et al., 2015). Under these conditions, the modelling assumptions match those used to arrive at the quantification formula in Alsop et al. (2015)^8^, although the formula is not used directly.

### 3.4 Macrovascular contamination

Macrovascular contamination arises due to the presence of labelled blood-water within major arteries at the time of imaging that is destined for brain tissue outside the voxel, and which causes an artificial increase in estimated perfusion within that voxel. This can be indicative of arterial transit artefacts, examples of which are given in Jaganmohan et al. (2021). Contamination from major arteries can be corrected using an extra component in the kinetic model (Chappell et al., 2010). Although contamination can arise with either single- or multi-delay labelling schemes, it can only be corrected for with multi-delay data. This is because the separation of macrovascular and perfusion signals relies on the different kinetics and arrival times of these two signal contributions that can only be observed when multiple delays are sampled. In practice, major macrovascular contamination is present in only a subset of voxels; invoking an extra component in the model increases the risk of overfitting and increases the uncertainty of perfusion estimates in voxels with no contamination. Hence, the magnitude of the macrovascular component in the model is subject to a shrinkage (or Automatic Relevancy Determination) prior (Mackay, 1995) that seeks to ensure that this component is only included where the data support it. This enhances the robustness of perfusion quantification across subjects that may have differing extents of macrovascular contamination (particularly if cerebrovascular disease is present).

If the data have calibration, the magnitude of the macrovascular component gives a measure of arterial blood volume (aBV) (Chappell et al., 2010; Petersen et al., 2006), the fraction of the voxel occupied by macro vessels, which are typically arterial. This should not be confused with the (total) blood volume, as estimated by other perfusion modalities that includes all vascular compartments in the voxel. The macrovascular component in the kinetic model follows the form of the AIF and thus can incorporate the effects of dispersion. The macrovascular component has a separate and independent arrival time parameter, the bolus arrival time (BAT), rather than the ATT which applies to the kinetics of the tissue.

### 3.5 Partial volume correction

A major feature of BASIL is the inbuilt partial volume effect correction (PVEc) method. PVEc seeks to separate the perfusion contributions from grey and white matter (and account for the zero-perfusion signal contribution from CSF) within a single voxel using estimates of the partial volumes of grey and white matter. The approach taken in BASIL follows the method in Chappell et al. (2011) whereby the signal in a voxel is modelled as a combination of grey and white matter kinetic signals which are mixed in proportion to the volume of each tissue within the voxel. Since this model is ill-posed, regularisation is imposed in the form of spatial priors on the grey and white matter perfusion values separately. This has the effect of using immediate neighbouring voxels to inform the tissue-specific perfusion estimates in a given voxel, following the principles used in other voxelwise correction methods (Asllani et al., 2008; Liang et al., 2013). Since this regularisation is a prior-based approach, it is adaptively driven by data quality, meaning that greater spatial detail can be preserved where the data support this (a detailed investigation is given in Zhao et al. (2017)). For example, where multi-delay data are used, which provides greater separability between grey and white matter kinetics due to differences in ATT and T1, the influence of the spatial prior will be automatically reduced. Although the method in Chappell et al. (2011) was originally demonstrated for multi-delay data, the implementation in BASIL generalises to the single-delay case (benefitting from specification of a prior on the noise parameter where only a few measurements are available).

PVEc requires voxelwise estimates of both GM and WM partial volumes. The pipeline can extract these from the output of an existing anatomical analysis using the FSL comprehensive anatomical analysis tool, fsl_anat; or by applying the FSL FAST segmentation tool (Zhang et al., 2001) to a supplied structural image; or by using user-supplied partial volume estimates directly. Estimates not in the same space as the ASL data (and particularly where the resolution of the estimates is higher than the ASL resolution, as is common for those derived from structural images) are transformed into ASL space using FSL applywarp with supersampling. In contrast with standard interpolation directly to the lower resolution, an intermediate supersampling step can be thought of as measuring the degree of overlap between corresponding voxels at the input and output resolutions, which better preserves partial volume data. The effectiveness of PVEc depends on the accuracy of partial volume estimates and, by extension, the accuracy of registration where this is needed to transform the estimates into the ASL data space (see Zhao et al., 2017).

When the BASIL pipeline is run with the PVEc option, it will do a normal, non-PVEc, analysis first and use this to initialise the PVEc analysis (the non-PVEc perfusion image is also used to refine registration with the structural image). In the final output, separate GM and WM perfusion images are produced, along with ATT images for multi-delay data. The algorithm calculates perfusion (and ATT) values for all voxels within the brain mask which will include extrapolation of values within voxels with little or none of the appropriate tissue. Hence, GM and WM masks (thresholded at 10% tissue partial volume) are used to produce masked GM and WM perfusion (and ATT) maps for visualisation and further analysis. As FAST partial volume estimates for subcortical structures cannot be interpreted in the same way as for cortical GM (due to differing tissue properties), these regions are removed from the PVEc output using the definitions of cortical grey matter and cerebral white matter in the Harvard-Oxford atlas. Perfusion estimates for these regions are still available in the non-PVEc output, but they are excluded from PVEc output because it is difficult to interpret them in light of the ambiguity of their partial volume estimates. Where this is not desired, the user will need to produce and apply their own ROI masks.

### 3.6 Calibration

The BASIL pipeline supports two widely used approaches to calibration:

- voxelwise, where the voxel values in the M0 image are used to estimate a magnetisation of arterial blood for each corresponding voxel in the perfusion image. This is the recommended approach of the ASL consensus paper and is more commonly used in clinical contexts (Alsop et al., 2015).
- reference region, where the mean intensity within a specific region of interest (ROI) of the M0 image is used to estimate a single global value for the magnetisation of arterial blood (Pinto et al., 2020).

**Fig. 5.**
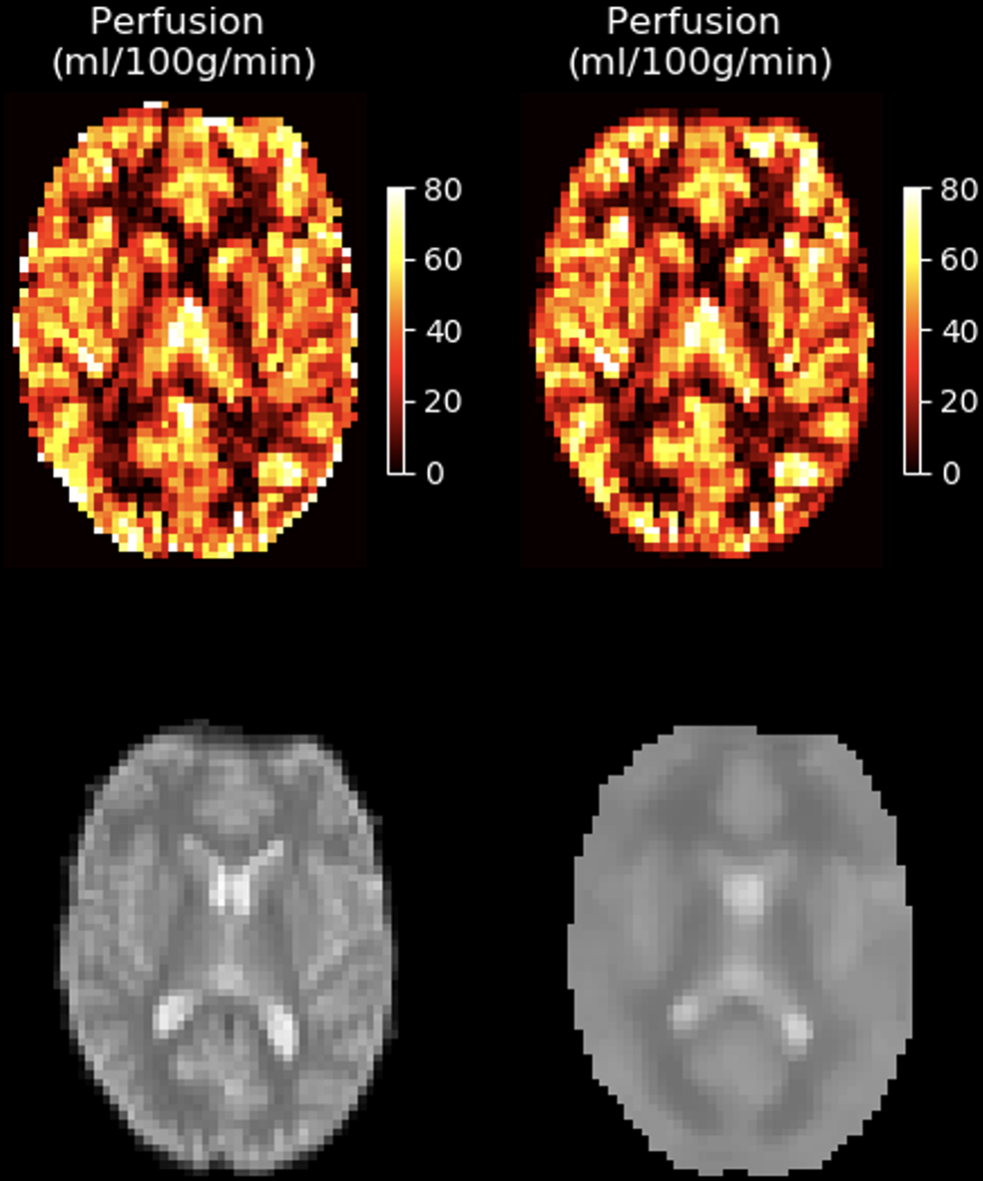
Calibration using a voxelwise approach. Left: Calibration using the original calibration image (lower image) results in spurious high perfusion voxels around the pial boundary of the brain. Right: Pre-processing of the calibration image (lower image) reduces the presence of CSF in pial voxels of this image, which suppresses artefacts in the resulting perfusion image.

For the voxelwise method, the toolbox follows the recommendations of the ASL consensus paper and corrects for proton density differences between tissue and blood using a relative water density (partition coefficient) of 0.9 (Herscovitch & Raichle, 1985), and corrects for a short TR when the TR for the M0 image is less than 5 s, assuming for all tissues a T1 of 1.3 s (the reference value for GM at 3 T). The calibration image is smoothed with a median filter (using a 3x3x3 voxel kernel) to suppress noise. To reduce “edge-effects” at the pial boundary, which arise due to partial voluming of brain tissue with CSF and tissue that are outside of the brain and give rise to a high intensity rim in the calibrated perfusion image, the pipeline implements a strategy of erosion (using a 3x3x3 voxel kernel) and extrapolation on the brain-masked M0 image prior to calibration. This pre-processing and the effect on the resulting perfusion image are illustrated in Figure 5. The derivation of the registration between the M0 image and ASL is discussed in the following section.

For the reference region method, the pipeline requires an ROI to be specified. By default, the ventricular CSF space is used, since this is an easy brain region to identify that will enclose multiple voxels without partial voluming at typical ASL resolution. Correction is made for partial T1 recovery and optionally for T2 mismatch between CSF and brain tissue using parameters in the Supplementary Material. Alternatively, a WM ROI can be specified, and appropriate tissue-specific corrections will be performed. A GM ROI can be provided but is not recommended due to partial volume effects. The pipeline includes the functionality to automatically generate the reference region ROI for the different tissue types should a structural image be available. The preference is to supply a structural image that has already had structural processing performed (including brain extraction and segmentation to generate partial volume estimates); to this end, the toolbox accepts the output of fsl_anat. Alternatively, FSL FAST will be applied directly to the brain extracted structural image to generate partial volume estimates with three tissue classes. When performing reference region calibration, it is not necessary to register the M0 image to the ASL.

For automated identification of a ventricular CSF ROI, the partial volume estimate for the CSF component from the FAST segmentation is selected; this is then masked with an ROI defined from the left and right ventricles from the Harvard-Oxford Atlas. The ventricular ROIs are transformed into the same space as the CSF partial volume estimates, thresholded at 0.1, binarised, and then eroded. The resulting masked CSF partial volume estimates are then transformed into ASL space using the registration performed between ASL data and structural image (using the downsampling process described in section 3.5). Finally, the resulting masked partial volume estimates are thresholded at 0.9 to leave only voxels with minimal partial volume effects. This procedure is deliberately conservative and is not meant to produce an accurate mask of the whole of the ventricular space, but rather to ensure a sample is taken from multiple voxels that are well within the ventricles.

For automatic definition of a WM ROI, the partial volume estimates from the WM component of the segmentation are transformed into the ASL data space (using the result of the registration to the structural image) and thresholded at 0.9; no further masking is applied.

Correction for coil sensitivity, where not performed during acquisition, can optionally be applied as part of the reference region calibration operation (it is implicit in the voxelwise method). This is achieved by either supplying a sensitivity image or indicating that the bias field of the structural analysis (fsl_anat) should be used, if available. Alternatively, two M0 images can be supplied, one of which is a reference with no (or minimal) sensitivity variation, typically acquired using the body coil.

### 3.7 Motion and distortion correction

Motion parameter estimation can optionally be performed using FSL MCFLIRT (Jenkinson et al., 2002) on the ASL timeseries with the M0 image as reference (if this is not possible, the middle volume in the series is used). The rationale for using the M0 image is to increase the robustness of the estimation to variation in images from ASL data in which the contrast varies, for example, due to variations in the static tissue signal present at different delays. To minimise interpolation artefacts when the estimated transformations are applied to the data (in case there is an overall motion related misalignment with the M0 image), the estimated transformations for each volume in the series are re-referenced to the middle volume in the series using the transformation between the middle volume and the calibration (which serves as the registration between ASL data and the M0 image, if required for voxelwise calibration). There exists some debate as to whether motion correction is advantageous for ASL imaging (Alsop et al., 2015) and it is left to the user to decide if it should be performed. One consideration is that high-motion volumes can be regarded as outliers in a model-fitting sense, and such outliers may introduce bias if they violate the underlying assumption of Gaussian noise. This is somewhat similar to approaches that apply variable weighting to the data: the algorithm will still tend towards the solution implied by the non-outlier data, but with greater uncertainty than would otherwise be the case. If motion correction is not requested, a registration between the calibration image and first volume of the ASL series is obtained using FSL FLIRT (and later updated once a perfusion image is available; see section 3.8).

The pipeline implements correction for distortion due to B_0_ field inhomogeneity when supplied either with a fieldmap, or an additional M0 image with reversed phase encoding compared to the main M0 image. When a fieldmap is supplied, FSL epi_reg is used to estimate the correction warp field which can then be combined with a user supplied gradient distortion warp field and applied to the ASL data series along with the estimated motion correction transformations. In this correction, the Jacobian of the warp is extracted and used to correct intensity scaling to account for the effects of distortion on signal intensity, though the correction is imperfect and SNR will remain lower in affected regions. When using phase-encoded reversed M0 images, FSL topup (Andersson et al., 2003) is used to estimate and apply distortion correction to data that have already had motion correction applied.

### 3.8 Registration

The registration functionality of the pipeline is built on FSL tools, specifically FLIRT (Jenkinson et al., 2002; Jenkinson & Smith, 2001) and epi_reg. The main objective of registration in the toolbox is to align the perfusion image (and other images in ASL acquisition space, such as ATT) with the structural image. The estimated transformation can be combined with others, such as the (non-linear) transformation between structural and MNI152 standard images provided by fsl_anat.

An approximate registration is performed both as an initialisation for the main registration and also for use in ASL data pre-processing prior to kinetic model fitting. For example, the creation of a brain mask from the anatomical image to define the extent of the analysis region in the kinetic model inference. This uses a 6 degree-of-freedom rigid FLIRT registration with either the brain extracted M0 image or mean of the label-control subtracted ASL timeseries as the base image.

The main registration process in the pipeline (performed by asl_reg) uses boundary-based registration (BBR) (Greve & Fischl, 2009) and thus needs to be provided with a white matter segmentation (normally obtained from the fsl_anat output). When a fieldmap is available, this can be included in the registration process (internally using the epi_reg command). By default, the pipeline uses the uncalibrated perfusion-weighted image, that is, after kinetic model fitting and before calibration, as the source image for this because it provides better contrast between grey and white matter than the control or M0 images, which is beneficial for BBR. The user can specify alternative registration sources: the mean difference image, the calibration image (if it is pre-registered with the ASL), or some other arbitrary reference. If major disturbances are expected, the user is recommended to perform their own registration and pass this directly to override all pipeline registration.

### 3.9 ROI reporting

When a structural image is provided, the pipeline will automatically report on the mean whole-brain perfusion within grey and white matter. For this, ROIs are defined from the partial volume estimates transformed to the resolution of the perfusion image and using a threshold of 90% for WM and 80% for GM. The lower threshold for GM is a pragmatic choice reflecting the low number of “pure” voxels at a typical ASL resolution, but the user can select a different threshold if appropriate for their data. For example, Chappell et al. (2021) suggest a threshold of 70% as a pragmatic choice for typical ASL resolution, for example, when following the resolution recommendations in Alsop et al. (2015). When available, these ROIs are also applied to calculate separate mean whole-brain GM and WM perfusion and ATT values after PVEc. A more restrictive cerebral GM value is also calculated by using the cortical GM and cerebral WM regions in the Harvard-Oxford atlas to mask out subcortical structures from the PVEc output maps.

Optionally, the BASIL pipeline will calculate summary measures of perfusion (and ATT where available) within ROIs defined by the Harvard-Oxford cortical and subcortical atlases. The probability maps from the atlases are transformed to the resolution of the perfusion images and thresholded at a probability fraction greater than 0.5. For any ROI with greater than 10 voxels, the following summary statistics are calculated: mean, standard deviation, median, and interquartile range. Additionally, the precision-weighted mean is calculated using the voxelwise precision (1/variance) estimates on the perfusion values. This measure thus accounts for variation in the confidence of perfusion estimates within the ROI. Supporting this, the I^2^ measure is also calculated, which describes the percentage of variation across voxels that is due to heterogeneity rather than chance (Higgins et al., 2003). Qualitatively, this indicates the variation of perfusion within the ROI that is not attributable to the estimated uncertainty in the voxelwise values. This is offered as a potentially useful metric, but it has not been explored extensively and no specific recommendation is made for its use. All of these summary measures are provided for the regions defined in the Harvard-Oxford atlases irrespective of the tissue content of the ROI, along with separate calculations where only the GM (at least 80% PV) or WM (at least 90% PV) are included.

### 3.10 Quality control

The BASIL toolbox does not currently provide any form of automated quality control (QC) for the main processing steps of the analysis pipeline, though this is an area of active research. A number of pipeline outputs permit the user to perform manual QC. These include summary measures of perfusion and ATT as detailed in section 3.9; perfusion estimates both before and after calibration; and the global M0 value or reference region mask used for calibration. The latter two help check for calibration issues and permit the user to perform their own calibration. When the pipeline is run with all structural processing options, pipeline outputs will be provided in native acquisition, structural and standard (MNI152) space, which enables the user to check registration quality.

## 4 Summary of Tools in the Basil Toolbox

asl_gui—the GUI for the toolbox. This offers a complete analysis solution for common ASL variants appropriate to the majority of use cases. This performs, via oxford_asl, the processing pipeline detailed in section 3. This article has largely focused on the processing steps available in the GUI.

oxford_asl—the main command line interface for the toolbox, which provides a scripting-based solution suitable for the majority of use cases. As with the GUI, it performs the processing pipeline detailed in section 3. In contrast to the GUI, oxford_asl also allows for greater user control over individual processing steps, and batch-processing of analyses prepared using the GUI.

The following tools are components of the BASIL toolbox that are used within oxford_asl and can be directly accessed by an advanced user building a bespoke ASL processing pipeline.

basil—the command line tool for the kinetic model inference, also incorporating PVEc. This allows for a variety of custom kinetic modelling to be performed on data, separate from other associated steps such as calibration and registration. This would be appropriate for a user who wishes to customise their kinetic analysis beyond the options available through oxford_asl, or wants to undertake that stage of analysis entirely independent of calibration and other processing performed using FSL tools.

asl_calib—a command line tool for performing the steps involved in calibration, namely the estimation of the magnetisation of arterial blood from an M0 image. This would be appropriate for a user who needs to perform a customised calibration, for example, using saturation recovery images, that is not offered by oxford_asl.

asl_reg—a command line tool that performs the steps needed for registration of ASL data to an anatomical image. This is a wrapper for other FSL registration tools (FLIRT and epi_reg) specifically tuned for ASL. This might be used if the default registration within oxford_asl is not successful for a given dataset.

asl_file—a command line helper tool for manipulating ASL data, this tool understands that ASL data come with combinations of label-control pairs and different delays within a single 4D image. This might be used to manipulate ASL data, for example, separate label and control images, perform subtraction, and undertake decoding of time-encoded data.

## 5 Associated and Related Tools

The BASIL toolbox contains a number of additional tools not included within the default pipeline implemented by oxford_asl, but which might be used where the data or application demands it.

asl_deblur—a command line tool that compensates for through plane blurring introduced in data with long readout out duration (such as single shot spiral or GRASE type acquisitions), based on the method used in Chappell et al. (2011).

enable—a command line tool for the automatic removal of low-quality or artefactual ASL data volumes, based on Shirzadi et al. (2017). This can be called from within oxford_asl.

fabber—a command line tool that performs non-linear model inference via the fast variational Bayesian inference algorithm from Chappell et al. (2009), including spatial priors (Groves et al., 2009; Penny et al., 2004). It is used within the BASIL toolbox for kinetic model inference, where it is called via basil command line tool. Within FSL, a variant of fabber is also offered for use with dual-echo ASL for functional MRI applications. The majority of users will not need to interact with this tool directly for ASL applications unless they wish to implement a different kinetic model or further customise parameter prior distributions.

FIX—a command line tool for ICA denoising of fMRI data that can be applied to ASL data (Carone et al., 2019).

quasil—a version of the basil command line tool tailored for QUASAR ASL that exploits the combination of flow-suppressed and non-suppressed data (Chappell, Woolrich, Petersen, et al., 2013; Petersen et al., 2006).

toast—a version of the basil command line tool tailored for Turbo-QUASAR data.

## 6 Future Directions

The BASIL toolbox was originally developed in the context of neuroimaging studies that focus on cortical GM perfusion and analysis of volumetric perfusion images. There is growing interest in accurate and robust measurements of perfusion in other brain regions and in other representations that are more specifically tied to the underlying anatomy. In the future, we intend for BASIL to support estimation of perfusion on the cortical surface (Kirk, 2021), exploiting information that is not available from a simple post-projection of volumetric perfusion onto the cortical surface, but instead using methods that account for partial volume effects around the cortex and can separate cortical GM perfusion from WM perfusion contributions. Perfusion images from the BASIL toolbox already include WM regions with specific WM perfusion estimates being produced via PVEc and mean WM perfusion being reported. In the future, the toolbox will additionally report on the perfusion within subcortical structures directly, incorporating knowledge of partial volume effects to make more accurate and structure-specific measurements.

Some of the design decisions and assumptions made in the toolbox may not be applicable for the study of disease. Tailoring of the toolbox for disease states is a major undertaking that has not been performed to-date and no claims are made to this effect. One particular area for consideration would be whether the priors could be updated with disease-appropriate values, though the purpose of priors is that the data can override them when reality is different to what the prior assumes, so pathology should appear in the results. The only time this breaks down is if the data are so noisy that they do not support any deviation from the prior. In Chappell et al. (2011), it was observed that the spatial prior could handle reasonably sharp changes in perfusion, for example, due to a lesion, without completely masking pathology.

For the oxford_asl pipeline specifically, two areas of future development concern automated QC and improved reporting. The goal with automated QC is to spot common failure modes for ASL analysis such as excessive motion, poor registration, and spurious calibration. For motion, a variety of strategies may be adopted, including frame censoring or variable weighting of the timeseries (Shirzadi et al., 2017; Tanenbaum et al., 2015). For reporting, the objective is to produce rich HTML documents with embedded figures and graphics for visual inspection of key pipeline outputs, as opposed to the textual-only reporting of the current version. A dedicated interface between the BASIL toolbox and the ASL-BIDS standard to enable batch processing of large datasets is in preparation.

## 7 Conclusion

The BASIL toolbox enables flexible and advanced analysis of ASL data in the brain with a focus on the quantification of perfusion and other haemodynamic measures. The toolbox is built around a Bayesian model-based inference algorithm. This allows it to be used on a wide variety of ASL data, allowing the user to exploit the advantages offered by multi-delay ASL variants, whilst also being able to process data from more commonly available acquisition protocols. The BASIL toolbox is an integrated part of FSL, allowing it to be used with other neuroimaging data and be integrated into multi-modal neuroimaging analysis pipelines.

## Data and Code Availability Statement

The BASIL toolbox is distributed as part of FSL: https://fsl.fmrib.ox.ac.uk/fsl/fslwiki/. The source code for the analysis pipeline (oxford_asl and Asl_gui) described in this paper can be found at https://github.com/physimals/oxford_asl. Quantiphyse is a GUI package aimed at non-specialist users that use BASIL: https://quantiphyse.readthedocs.io/en/latest/.

## Author Contributions

Michael A. Chappell: Conceptualisation, Methodology, Software, Writing—Original Draft, and Supervision; Thomas F. Kirk: Software, Writing—Original Draft; Martin S. Craig: Software, Writing—Review & Editing; Flora A. Kennedy McConnell: Software, Validation, and Writing—Review & Editing; Moss Y. Zhao: Methodology, Software, and Writing—Review & Editing; Bradley J. MacIntosh: Methodology, Software, and Writing—Review & Editing; Thomas W. Okell: Conceptualisation, Methodology, and Writing—Review & Editing; and Mark W. Woolrich: Conceptualisation, Writing—Review & Editing, and Supervision.

## Funding

This work was supported by the Engineering and Physical Sciences Research Council UK (EP/P012361/1). Thomas W. Okell was supported by a Sir Henry Dale Fellowship jointly funded by the Wellcome Trust and the Royal Society (Grant Number 220204/Z/20/Z). Flora A. Kennedy McConnell is supported by the Beacon of Excellence in Precision Imaging, University of Nottingham. Moss Y. Zhao is supported by the American Heart Association (Grant #826254) and National Institutes of Health (Grant R01EB025220-02). Mark W. Woolrich’s research is supported by the NIHR Oxford Health Biomedical Research Centre, the Wellcome Trust (098369/Z/12/Z, 106183/Z/14/Z, 215573/Z/19/Z), and the New Therapeutics in Alzheimer’s Diseases (NTAD) study supported by UK MRC and the Dementia Platform UK. The Wellcome Centre for Integrative Neuroimaging is supported by core funding from the Wellcome Trust (203139/Z/16/Z).

## Declaration of Competing Interest

Michael A. Chappell & Mark W. Woolrich receive royalties for commercial licensing of FSL. Michael A. Chappell & Thomas F. Kirk are employed by and hold equity in Quantified Imaging Ltd.

## Supplementary Material

Supplementary Material

## Footnotes

1 A pubmed search with “Arterial Spin Labeling MRI” returns over 300 publications per year since 2015.

2 It is possible to update BASIL within a prior release of FSL v6 to the specific version described here without updating the whole FSL package.

3 Literature search carried out in May 2023 based on citations for Chappell et al. (2009), limiting only to papers including the term Arterial Spin Labelling, excluding self-citations and publications that report new ASL techniques or methods comparisons.

4
https://docs.google.com/document/d/e/2PACX-1vQ-1GF2fmz6Q4IukuKP_-57H-xi872Xq_uBlX5P0Cwpj4RYd_t73pvZ64UqXegPaVpQJhQQrVRJRPro/pub

5
https://www.nitrc.org/plugins/mwiki/index.php/dcm2nii

6 If alignment to anatomical or standard space is required, the pipeline transforms the perfusion estimates rather than the ASL data.

7 Formally, the noise precision parameter has as its posterior distribution a gamma distribution with two parameters and is subject to a gamma distribution prior.

8 Namely, identical tissue and blood T1 values, and no venous outflow component.
