# Supplementary material for: BASIL: A toolbox for perfusion quantification using arterial spin labelling
Source: Imaging Neurosci (Camb). 2023 Dec 5;1:imag-1-00041. doi: 10.1162/imag_a_00041 (PMC12007520; doi:10.1162/imag_a_00041)
Supplement: Supplementary Material [file imag_a_00041-supp.pdf]

BASIL: A Toolbox for Perfusion Quantification using Arterial Spin Labelling

Supplementary Material

Setting the noise prior when there are a small number of volumes

When there are fewer than 5 volumes in the input data the use of a non-informative prior on the noise parameter could lead to poor algorithmic convergence. For this case we implement a more informative prior. Taking an SNR of 10 we calculate an equivalent noise magnitude for the data by scaling with an estimate of the perfusion signal magnitude. This is required since we cannot know a priori what the intensity scale will be for arbitrary (uncalibrated) ASL data. The label-control subtracted data at each delay is averaged and the maximum value at each voxel over all the delays extracted. This is averaged across the brain (excluding zero voxel values) and scaled by 2.0 to reflect the fact that this will be a mixture of GM, WM and CSF; and so will be approximately half of the mean GM perfusion.

Model parameter prior distributions

Table S1 Specification of parameter priors in BASIL, each parameter is described by a normal distribution with mean and precision, the equivalent standard deviation is also given here for reference. T1 values are based on a 3T field strength; the user should provide values for other field strengths. ATT = Arterial Transit Time, aBV = arterial Blood Volume, BAT = Bolus Arrival Time

Parameter	Mean	Precision	Std. dev.
Tissue (grey matter)			
Perfusion (relative)[a]	0	10^{-6}	1000
ATT	1.3 | 0.7[b]	1.0[c]	1.0
T1	1.3	100	0.1
Partition co-efficient [d]	0.9 (0.98)	-	-
Arterial blood/Macrovascular component			
aBV	0	*variable*[e]	
BAT	ATT_{GM} - 0.3	1.0 [c]	1.0
T1b	1.65	100	0.1
White matter			
Perfusion[a]	0	10^{-6}	1000
ATT	ATT_{GM} + 0.3	1.0 [b]	1.0
T1	1.1	100	0.1
Partition co-efficient [d]	0.82	-	-

[a] It is assumed that the tissue perfusion parameter in the kinetic model is relative (and that calibration will be performed after kinetic model inference). This prior is uninformative, hence has a very small precision compared to the typical image intensity values (~1-100).

[b] The mean of the prior for ATT is set based on the labelling used, PASL uses 0.7 s, PCASL (and CASL) 1.3 s. These are based on experience with these types of labelling, but can be adjusted by the user where their acquisition might produce typically longer or shorter ATT based on position of labelling region/plane.

[c] For single-delay data the precision on the ATT parameters is set to 10 (standard deviation of 0.316). The choice of precision reflects a reasonable range of typical ATT within approximately one standard deviation.

[d] For a generic tissue compartment a combined partition coefficient value is used (0.9), when modelling both grey and white matter separately (for PVEc) individual values are used (Herscovitch and Raichle, 1985), i.e., 0.98 for GM and 0.82 for WM.

[e] A shrinkage prior is used and thus the precision is inferred from the data.

Residue and arterial input functions

Table S2 Residue functions and arterial input functions (AIF) implemented in BASIL. All combinations for both PASL and PCASL labelling are possible as indicted by a ○ in the relevant entry, for specific combinations an analytic solution has been implemented for the convolution required to define the tissue kinetics, indicated by ●, otherwise a numerical convolution is used.

**Residue function**	Single-compartment			Two-compartment	
**AIF**	Simple	Well-mixed[a]	impermeable	Parkes & Tofts	Single pass approximation
Box-car (no dispersion)	●	●	●	●	●
Gamma variate function	○	○	○	○	○
Dispersion kernel					
Gaussian	○	○	○	○	○
Spatial Gaussian	○	○	○	○	○
Gamma	○	●	○	○	○

[a] For the single well-mixed compartment, in the residue function the perfusion parameter that appears in the outflow term is assumed to be 0.01 s^{-1} (60 ml/100g/min), unless it is specifically indicated that the data has already been calibrated in advance.

Calibration tissue parameters

Table S3 Tissue parameters used in calibration.

Parameter	Grey matter	White matter	CSF
T1 (s)	1.3	1.0	4.3
T2/T2* (ms)	100/60	50/50	750/400
Partition co-efficient	0.98	0.82	1.15
